# The entomopathogenic fungus *Beauveria bassiana *reduces instantaneous blood feeding in wild multi-insecticide-resistant *Culex quinquefasciatus *mosquitoes in Benin, West Africa

**DOI:** 10.1186/1756-3305-3-87

**Published:** 2010-09-15

**Authors:** Annabel FV Howard, Raphael N'Guessan, Constantianus JM Koenraadt, Alex Asidi, Marit Farenhorst, Martin Akogbéto, Matthew B Thomas, Bart GJ Knols, Willem Takken

**Affiliations:** 1 Laboratory of Entomology, Wageningen University and Research Centre, P.O. Box 8031, 6700 EH Wageningen, The Netherlands; 2 London School of Hygiene and Tropical Medicine, Keppel Street, WC1E 7HT, London, UK; 3 Centre de Recherche Entomologiques de Cotonou (CREC), 06 BP 2604 Cotonou, Benin; 4 Center for Infectious Disease Dynamics and Department of Entomology, Penn State University, 1 Chemical Ecology Lab, University Park 16802, PA, USA; 5 Div. Infectious Diseases, Tropical Medicine & AIDS, Academic Medical Center, F4-217 Meibergdreef 9, 1105 AZ Amsterdam, The Netherlands

## Abstract

**Background:**

Mosquito-borne diseases are still a major health risk in many developing countries, and the emergence of multi-insecticide-resistant mosquitoes is threatening the future of vector control. Therefore, new tools that can manage resistant mosquitoes are required. Laboratory studies show that entomopathogenic fungi can kill insecticide-resistant malaria vectors but this needs to be verified in the field.

**Methods:**

The present study investigated whether these fungi will be effective at infecting, killing and/or modifying the behaviour of wild multi-insecticide-resistant West African mosquitoes. The entomopathogenic fungi *Metarhizium anisopliae *and *Beauveria bassiana *were separately applied to white polyester window netting and used in combination with either a permethrin-treated or untreated bednet in an experimental hut trial. Untreated nets were used because we wanted to test the effect of fungus alone and in combination with an insecticide to examine any potential additive or synergistic effects.

**Results:**

In total, 1125 female mosquitoes were collected during the hut trial, mainly *Culex quinquefasciatus *Say. Unfortunately, not enough wild *Anopheles gambiae *Giles were collected to allow the effect the fungi may have on this malaria vector to be analysed. None of the treatment combinations caused significantly increased mortality of *Cx. quinquefasciatus *when compared to the control hut. The only significant behaviour modification found was a reduction in blood feeding by *Cx. quinquefasciatus*, caused by the permethrin and *B. bassiana *treatments, although no additive effect was seen in the *B. bassiana *and permethrin combination treatment. *Beauveria bassiana *did not repel blood foraging mosquitoes either in the laboratory or field.

**Conclusions:**

This is the first time that an entomopathogenic fungus has been shown to reduce blood feeding of wild mosquitoes. This behaviour modification indicates that *B. bassiana *could potentially be a new mosquito control tool effective at reducing disease transmission, although further field work in areas with filariasis transmission should be carried out to verify this. In addition, work targeting malaria vector mosquitoes should be carried out to see if these mosquitoes manifest the same behaviour modification after infection with *B. bassiana *conidia.

## Background

Current mosquito control for the prevention of malaria and other vector-borne diseases relies heavily on pyrethroid insecticides, most notably through the use of insecticide-treated nets (ITN) and indoor residual spraying (IRS) [1]. Unfortunately, the emergence of insecticide resistance in some geographical areas is threatening vector control efforts [2]. It is widely accepted that the emergence of insecticide resistance in mosquitoes in Benin, as in many other areas of the world, was due to heavy pesticide use in agriculture [3-7]. However, the impact of this resistance is increasingly affecting the public health sector as well. It is therefore important to search for alternative tools that can be used to control insecticide-resistant mosquitoes.

The entomopathogenic fungi *Metarhizium anisopliae *and *Beauveria bassiana *can be used to target a wide range of insects [8,9] including adult mosquitoes [10-12]. In addition, these fungi can cause significant mortality to insecticide-resistant *Anopheles *mosquitoes in the laboratory [13-15], with insecticide-resistant mosquitoes being significantly more susceptible to fungal infection when compared to insecticide-susceptible mosquitoes [15]. This finding could lead to interesting possibilities with population dynamics because insecticide-resistant genes would be removed from the mosquito population at a faster rate and this would lead to the conservation of insecticide-susceptible genes.

The conidia of entomopathogenic fungi, once germinated, directly penetrate the mosquito cuticle. Once inside the mosquito haemocoel, the fungi produce compounds and eventually kill the mosquitoes by a combination of nutrient depletion and internal mechanical damage [16]. According to previous research, this starts leading to insect death approximately three-to-four days after infection [10,11,17]. This slow kill time is in contrast to fast acting insecticides currently in use and potentially allows fungus-infected mosquitoes to attain some of their life-time reproductive output, which could reduce selection pressure for resistance. Accordingly, in order to determine overall transmission blocking (and indeed, overall fitness costs of infection) it is important to evaluate not only the mortality rate but also sub- and pre-lethal consequences of infection. In this regard, previous studies have revealed reductions in mosquito feeding propensity [18] and fecundity [18] due to fungal infection, and *B. bassiana *was shown to limit the development of malaria parasites in the mosquito [19].

Due to proposed future application methods, it is important to test entomopathogenic fungi alone and in the presence of existing control tools such as ITNs so as to monitor any potential additive or synergistic effects [20], as were found in a recent laboratory study that showed that fungal and permethrin combination treatments can cause increased mosquito mortality when compared to either treatment alone [21].

Despite encouraging results from the laboratory, only two studies have been published using these entomopathogenic fungi against mosquitoes in the field, and neither of these studies targeted insecticide-resistant mosquitoes [22,23]. Both studies showed that fungal infections significantly shortened the life span of infected mosquitoes when compared to uninfected mosquitoes [22,23], but neither study examined behavioural effects such as blood feeding.

In this study an experimental hut trial was conducted in Benin, West Africa, to assess whether wild multi-insecticide-resistant mosquitoes would be infected by *M. anisopliae *or *B. bassiana *when applied to window netting. These fungal treatments were evaluated in the presence of an untreated or permethrin-treated bednet. We examined mortality, effect on blood feeding and other behaviours such as deterrence and induced exophily.

## Materials and methods

### Mosquitoes

The mosquitoes used in the behaviour experiments in the laboratory were *An. gambiae s.s. *VKPER. This is a pyrethroid-resistant strain that was initially collected from the Valley du Kou in Burkina Faso and then selected repeatedly to fix the *kdr *gene. This gene is linked to knockdown resistance to pyrethroids and DDT. For the laboratory experiments, eggs from a colony maintained at the Centre de Recherche Entomologique de Cotonou (CREC) in Benin, were brought to Wageningen University in The Netherlands and a colony was started. Mosquitoes were subject to standard rearing using tap water in plastic trays (10 × 25 × 8 cm) and fed with 'Tetramin^®^' fish food daily. Pupae were selected daily and adults were held in standard 30 × 30 × 30 cm cages and fed on a 6% glucose solution *ad libitum*. The larval trays and adult cages were kept in climate chambers held at 27°C (± 1), 80% RH (± 10) and a 12:12 hr L:D photoperiod.

At the field site (described below) in Benin, West Africa, the wild *An. gambiae *population has been shown to be 100% *An. gambiae s.s. *Mopti cytotype [3]. *Culex quinquefasciatus *Say is also present and both species are resistant to pyrethroids, DDT and dieldrin [2,3,24,25]. Resistance mechanisms involve the *kdr *gene mutation, mixed function oxidase (MFO) and esterase levels that are higher than in reference susceptible strains [3]. In addition, *Cx. quinquefasciatus *is resistant to carbosulfan and has elevated glutathione-*S*-transferase (GST) activity [3].

### Fungi

We examined the effect of two fungal species. *Metarhizium anisopliae *var. *anisopliae *(Metsch.) Sorokin isolate ICIPE-30 was produced using solid state fermentation with glucose-impregnated hemp in 200 ml aerated packed tubes at Wageningen University, The Netherlands. *Beauveria bassiana *(Balsamo) Vuillemin IMI 391510 was produced by initially growing the fungus in a liquid medium and then inoculating autoclaved barley flakes in mushroom spawn bags at Penn State University, USA.

After being dried at ambient temperature and then stored in the refrigerator, dry conidia of *M. anisopliae *and *B. bassiana *were separately suspended in the synthetic isoparaffinic hydrocarbon solvent ShellSol T ™ (Shell, The Netherlands). ShellSol T was selected because the delivery system of fungal conidia suspended in this solvent has been shown to be significantly more virulent to *An. gambiae s.s. *mosquitoes when compared to conidia suspended in other oils [26]. A Bürker-Türk haemocyte counter and light microscope (at ×400) were used to determine accurate conidial concentrations per ml ShellSol T. New suspensions were made for each experimental replicate. As a measure for conidial viability, the germination of spores on a rich agar medium was counted as previously reported [15]. The mean (± SE) viabilities of the batches of *M. anisopliae *and *B. bassiana *used in this study were 8.5% (± 2.62; 2339 spores counted) and 64% (± 4.70; 2381 spores counted) respectively.

### Net treatment with the fungal conidia

**Figure 1:**
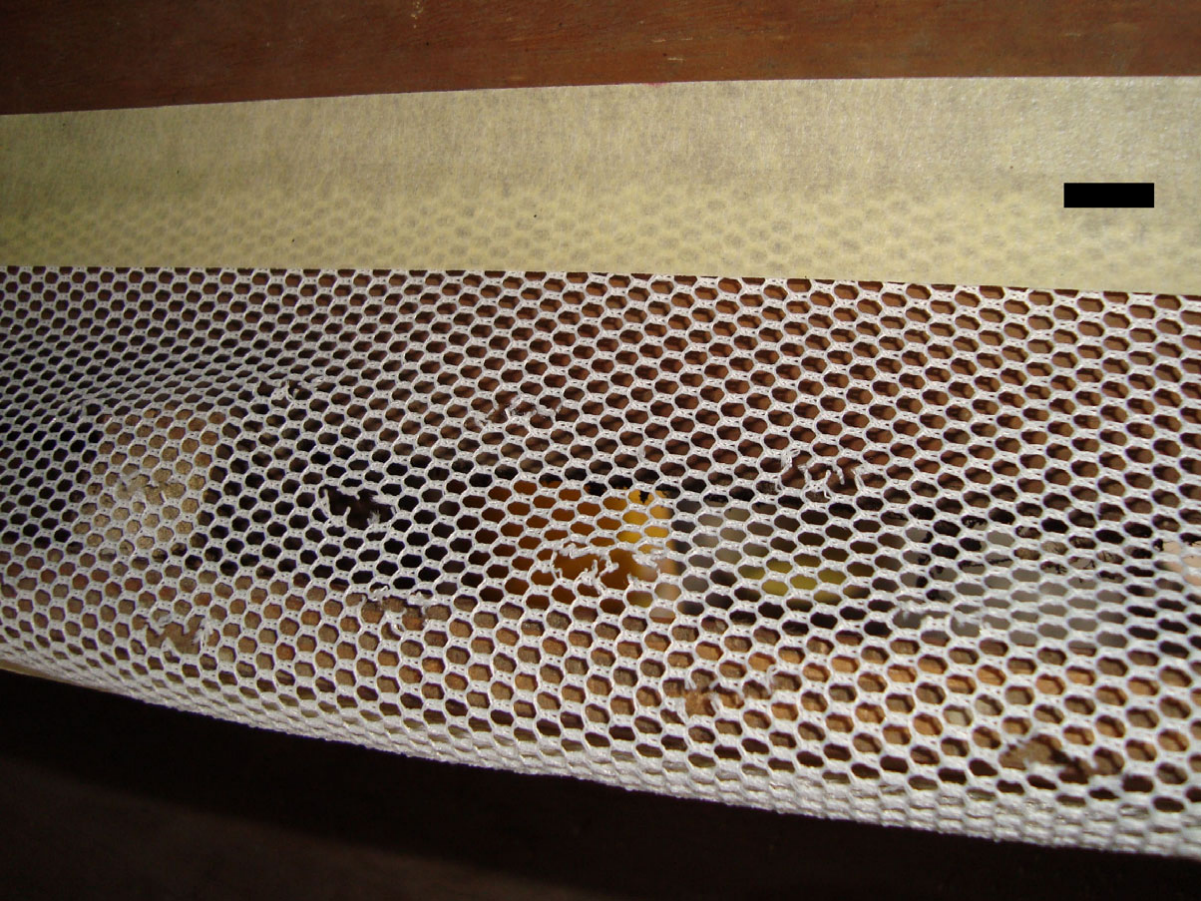
**A close up of netting attached to the inside of the experimental hut windows**. The slits cut to facilitate mosquito passage through the netting can be seen, the scale bar (top right) represents 1 cm

The netting used was made of white 100% multifilament 150 denier warp-knitted polyester fibres with 12 holes per cm^2 ^(Vestergaard Frandsen, Switzerland). This net was used to cover the windows in the experimental hut trials and for the behaviour experiments in the laboratory. Unpublished work found that around 50% of mosquitoes would pass this mesh-sized netting when a human host cue was provided on the other side, whilst fewer mosquitoes passed the smaller mesh-sized netting [27]. In an effort to increase the proportion of mosquitoes passing through the netting, small slits were cut into the netting to facilitate mosquito passage (Figure 1). Obviously, in a proper control setting we would not advocate damaging the physical integrity of window or eave screens but for this experimental test, it was necessary to allow mosquitoes access to the huts in order to sample them post-exposure. Netting was dipped into the fungal conidia/ShellSol T suspensions resulting in treatment densities of 7.2 × 10^12 ^conidia per m^2^. Control netting was treated with ShellSol T only.

### Behaviour experiments in the laboratory

**Figure 2:**
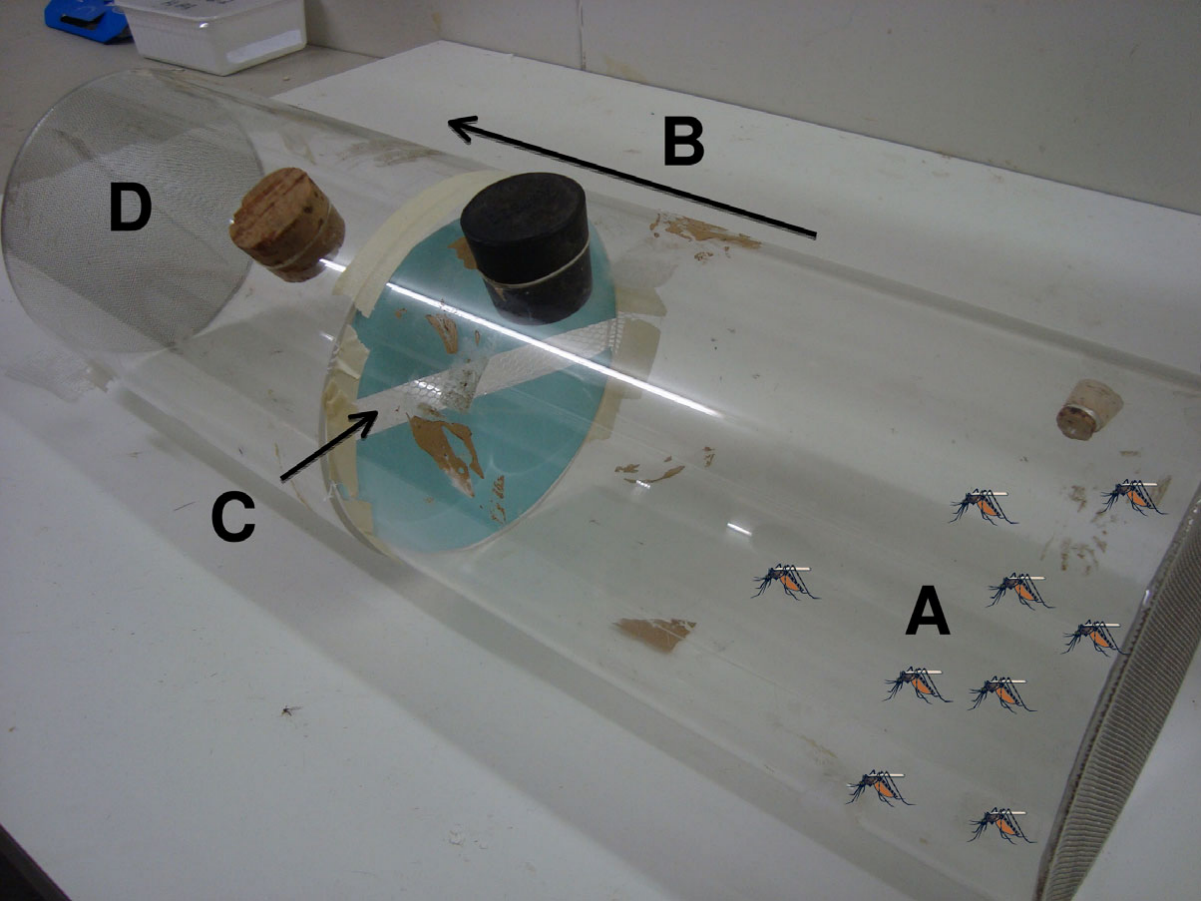
**The behaviour experiment apparatus used whilst monitoring the movement of mosquitoes through the slit netting in the laboratory**. Mosquitoes were introduced into one half of the cylinder (A), they move in the direction indicated (B), crossing the card with the 1 cm gap (C) until they reach the attractive odour sources (not shown but in position (D))

To test the suitability of fungus-treated nets for infecting hut-entering mosquitoes, and to confirm that mosquitoes would be able to pass through the netting, behavioural assays were conducted in the laboratory at Wageningen University. The experimental set-up (Figure 2) contained a transparent plastic cylinder (15 cm diameter × 50 cm length) with a separating piece of cardboard in the centre (C in Figure 2). A 1 cm slit was made lengthways in the centre part of the cardboard, representing the gaps of the windows in the experimental huts. The ends of the cylinder were sealed with wire netting to allow air to pass through. At one end, a heating element set between 33.5°C and 34.5°C, humid air and a worn nylon sock (not shown but in position D in Figure 2) were used to entice mosquitoes released at the other end of the cylinder (A in Figure 2) to pass through the 1 cm gap. A small amount of suction at the opposite end was used to move the odour through the cylinder. The test was carried out under a red light and started during the night so that the mosquitoes were more likely to initiate host seeking.

In the first set of experiments in one of the cylinders, the 1 cm gap in the cardboard was left clear, while in the second cylinder untreated white polyester netting was placed over the gap. Six-to-nine day old non-blood fed female *An. gambiae s.s. *VKPER strain mosquitoes were selected immediately before the test based on a response to a human hand; mosquitoes of this age were used because host seeking peaks at 6 days post emergence [28]. Twenty-five mosquitoes were placed into each tube at a time, such that they had to pass the gap or net to contact the heat and odour source. The test was run for 30 minutes; four replicates were carried out.

In the second set of experiments both cylinders had the slit-cut netting covering the cardboard gap; one cylinder was the control and in the other, the net had been treated with *B. bassiana *24 hours before the test began. Fifty 6-9 day old non-blood fed female *An. gambiae s.s. *VKPER mosquitoes were selected per replicate, with two replicates run per cylinder. The tests were run for 1 hour, after which time all mosquitoes that had passed/not passed the netting were removed and kept in cups and given access to 6% glucose solution *ad libitum*. After five days surviving mosquitoes were killed, dipped in 70% ethanol (to sterilize them externally) and placed onto moist filter paper in Petri dishes. These were then sealed with Parafilm and placed in a 27°C incubator in the dark. Three days later the proportion of the mosquitoes infected with the fungus was visually scored by checking the presence of sporulation/emerging hyphae. In this way, it was possible to determine the minimum proportion of the mosquitoes that had passed through the netting that had picked up a fungal infection. Similarly, we scored how many mosquitoes had contacted the netting, picked up an infection but had not passed through the net.

### Field study location and experimental hut design

**Figure 3:**
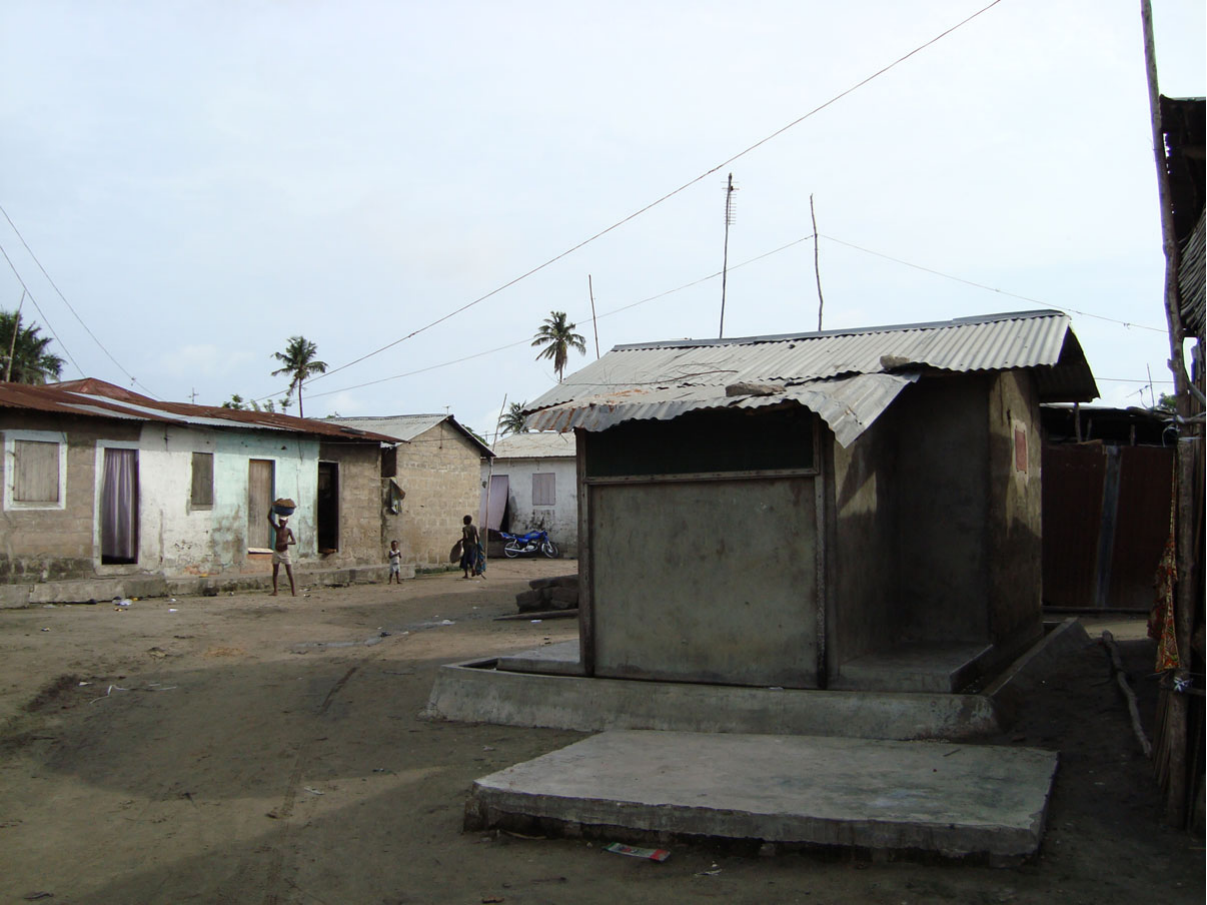
**One of the experimental huts (on the right) used for the trial**. These huts are located inside Ladji village and houses can be seen on the left

The experimental hut study was undertaken in Ladji village (6°23'23N, 2°25'56E) on the shore of Lake Nokoué in the northern outskirts of Cotonou, in Benin, West Africa. Concrete experimental huts have been built within this village (Figure 3) so that they more accurately represent the village dynamics with respect to mosquito house entry. These huts were of the typical West African design with corrugated iron roofs that do not have eaves (for a schematic representation see Hougard et al. [29]). The ceilings of the huts were thick polyethylene sheeting. Mosquitoes can only enter the huts through four windows. These windows were 60 cm long and consist of metal funnels that channel mosquitoes into a 2 cm gap. This means that once mosquitoes have entered the hut they are unlikely to leave via the windows. Mosquitoes wanting to leave the huts instead fly towards the large veranda trap which, being partial netting, is lighter than the hut interior. The huts are protected from ants by a water moat.

### Pre-intervention mosquito entry

The intention of the experimental hut trial was to use the window gaps of the experimental huts for the application of fungus-impregnated netting to target the entering mosquitoes with fungal spores. Therefore, before the field trial it was necessary to check whether wild mosquitoes would pass through the window netting. Three of the above-described huts had control netting attached to the inside of the funnelled windows such that every mosquito that entered the huts had to pass through the netting. In the other three huts, the windows were left uncovered. The netting and uncovered windows were then rotated between the huts. This preliminary trial was run for 17 nights from the 6^th ^to the 25^th ^April 2009.

### Hut treatments

By using treated netting across the opening of the funnel windows of the experimental huts (Figure 1) we ensured that all the mosquitoes entering the huts contacted the fungal spores. The fungus-treated netting was treated as described above. The control window netting was treated with ShellSol T. Netting was treated and attached to the inside of the window openings in the huts on the same day. For each of the six replicates, new pieces of netting were impregnated with freshly made fungal suspensions. The fungus treatment amounted to 0.13 m^2 ^per hut.

The bednets used were white 100 denier polyester netting (SiamDutch Mosquito Netting Co., Thailand) measuring 2.11 m length × 1.63 m width × 1.84 m high with a total surface area of 17.2 m^2^. Each bednet had six holes cut (4 cm × 4 cm) as recommended by the World Health Organisation (WHO) [30] to mimic worn bednets; this allows blood feeding behaviour to be monitored. Three nets were treated with permethrin 25EC (Syngenta, Switzerland) at 500 mg/m^2 ^and three others were left untreated to serve as controls. Untreated nets were used because we wanted to test the effect of fungus alone and in combination with an insecticide to examine any potential additive or synergistic effects between the fungi and permethrin treatments.

The six treatments (Table 1) were randomly allocated between six huts and then rotated weekly using a Latin square design such that each treatment spent one week in each hut. The hut trial was run for 36 nights between 27^th ^April and 6^th ^June 2009. A temperature and humidity gauge was left inside one of the huts for the duration of the trial.

**Table 1:** Experimental hut data showing the effects of fungal and insecticide treatment combinations on wild *Cx. quinquefasciatus*; significant p-values are in bold

Window Treatment	Bednet treatment	Code	N	Mortality at 7 days % (95% CI)	Blood fed % (95% CI)	BFI%	PP%	Blood fed OR (95% CI)	p
Control	Control	CC	207	53.6 (46.8,60.4)	30.4 (24.2,36.7)	-	-	-	-
Control	Permethrin	CP	167	50.9 (43.3,58.2)	17.4 (11.6,23.1)	42.9	19.3	0.53 (0.3,0.9)	**0.012**
*M. anisopliae*	Control	MC	177	50.8 (43.3,58.2)	31.6 (24.8,38.5)	-3.9	14.5	1.15 (0.7,1.8)	0.54
*M. anisopliae*	Permethrin	MP	127	52.8 (44.1,61.4)	15.0 (8.8,21.2)	50.8	38.7	0.58 (0.3,1.0)	**0.045**
*B. bassiana*	Control	BC	168	57.7 (50.3,65.2)	19.0 (13.1,25.0)	37.4	18.8	0.58 (0.4,0.9)	**0.032**
*B. bassiana*	Permethrin	BP	172	54.1 (46.6,61.5)	17.4 (11.8,23.1)	42.7	16.9	0.53 (0.3,0.9)	**0.012**

BFI = Blood Feeding Inhibition; PP = Personal Protection; OR = Odds Ratio

### Hut trial procedure

The study received ethical approval from the Ministry of Health, Cotonou, Republic of Benin, in May 2008 (approval n° 10717/MSP/DG/SGM/DRS). Six adult males from Ladji village were then recruited as sleepers after they had provided their informed consent to participate in the study. Malaria treatment was offered to these sleepers if they developed malaria during the trial. To control for individual attractiveness [31], the sleepers rotated between the huts nightly. During the evening the day after a new treatment had been placed in the huts, all the sleepers were individually asked a short series of questions to determine whether they had any health issues associated with the treatments. The questionnaire was carried out over six weeks such that all sleepers were questioned after sleeping under each treatment.

The external window shutters on the huts were opened at 6 pm and the sleepers entered the huts at 9 pm. At 5 am the following morning a curtain was unrolled to separate the veranda from the hut and at this time the sleepers collected all dead and live mosquitoes using a mouth aspirator. The mosquitoes from the hut, veranda trap and those found inside the bednet were kept in separate cups. The collected mosquitoes were identified to sex/species and the females were recorded as dead/alive and blood fed/unfed. Live mosquitoes were then held in plastic cups, given access to honey solution and mortality was scored every 24 hours [32]. For logistical reasons the mosquitoes that arrived in the laboratory alive from the huts were only able to be kept for a maximum of 7 days, after which time they were killed.

While monitoring the impact of the treatments on mosquito survival, a series of behavioural outcomes were also scored. When compared to mosquitoes collected from the control (Table 1; CC) hut it was possible to see whether any of the treatment combinations had caused blood feeding inhibition (smaller proportion of blood fed mosquitoes). Furthermore, if a treatment deters mosquitoes from entering the huts then the proportions that were blood fed may underestimate the full personal protective effect. This can be calculated using the following formula:

$$\%\text{ Personal Protection}=\text{1}00\left({\text{B}}_{\text{u}}-{\text{B}}_{\text{t}}\right)/{\text{B}}_{\text{u}}$$

where B_u _is the total number of blood fed mosquitoes collected from the untreated control hut and B_t _is the total number of blood fed mosquitoes collected from the treated hut [30]. In addition, any effects on deterrence (fewer mosquitoes entering the huts) and/or induced exophily (more mosquitoes entering the veranda trap) were measured.

### Statistical analysis

#### Behaviour experiments in the laboratory

Due to differences between the replicates with respect to the initial mosquito responsiveness, the behaviour experiment data were analysed 10 minutes after the first mosquitoes had passed the gap/netting. This is because it was observed that after this time the vast majority of the mosquitoes that were going to respond to the odour had already responded. Data on mosquito passage was analysed using Chi-square tests. Due to the low numbers of mosquitoes that did not become infected by the *B. bassiana *in experiment two, the Fishers exact test was used to analyse the difference in fungal infection rates between the mosquitoes which passed the net compared to those that did not pass.

#### Hut trial data

Blood feeding was analysed using binomial logistic regression. Statistical outcomes were given as Odds Ratios (OR) which gives the ratio of the odds of an event occurring in one group to the odds of it occurring in another group. The survival analyses of the mosquitoes collected from the huts were investigated using Cox regression analysis. Mortality rates were given as Hazard Ratios (HR), which give the average daily risk of dying relative to the control. Hut attractiveness, treatment deterrence, induced exophily and immediate mortality were separately analysed using single factor ANOVA analysis.

All statistics were carried out in SPSS 17.0 [33] with α at 0.05.

## Results

### Behaviour experiments in the laboratory

In the first set of experiments, there was no significant difference between the numbers of *An. gambiae s.s. *VKPER mosquitoes that passed the gap (48/100) compared to those that passed the net with slits cut into it (45/100) (χ^2 ^= 0.18, d.f. = 1, p = 0.67). Similarly, in the second set of experiments there was no significant difference between the number of *An. gambiae s.s*. VKPER passing either the control (59/96) or *B. bassiana *(57/103) treated net (χ^2 ^= 0.77, d.f. = 1, p = 0.38), indicating that this malaria vector is not deterred by the entomopathogenic fungus. Of the mosquitoes that passed the treated net, 98% (56/57) showed infection with *B. bassiana *after death, while 89% (41/46) of mosquitoes that did not pass the netting showed *B. bassiana *infection; this difference was not significant (Fishers exact test; p = 0.09).

These results showed that our proposed protocol for the field work, where mosquitoes were expected to pass through screened windows, should allow mosquitoes to enter the huts through the slit netting leading to fungal infection.

### Pre-intervention mosquito entry

Over the 17 pre-intervention nights in the experimental huts, 1356 mosquitoes were collected. Of the 1073 females, 86.7% were *Cx. quinquefasciatus *and 13.3% *An. gambiae s.l.*. When compared to the number of mosquitoes entering the huts without the netting, the untreated slit window nets reduced culicine female entry by 29% and anopheline female entry by 64%.

### Hut trial data

During the hut trial the temperature and humidity ranges were 25.1 - 36.4°C and 69 - > 95%RH respectively inside the huts, with daily means (± SE) of 30.8°C (± 0.23) and 84%RH (± 1.33). For each week the maximum recorded temperature and humidity was above 34°C and 95% RH respectively. Out of the 216 questions asked to the sleepers during the trial, no adverse effects (such as respiratory difficulties, skin irritation or headaches etc.) due to the fungal treatments were reported.

A total of 1955 mosquitoes were collected in the huts over 36 intervention nights; 1018 *Cx. quinquefasciatus *females, 87 *An. gambiae s.s. *females, 20 *Aedes aegypti *L. females and 830 males of several different genera. Only seven *An. gambiae s.l. *females entered our control (CC) hut during the six-week hut trial. The 64% reduced entry rate calculated during pre-intervention data collection indicates that only a predicted 19 *An. gambiae s.l. *would have entered the CC hut if there was no netting on the windows. This would still not have been enough to carry out adequate statistical analysis. Due to the low number of *An. gambiae s.s. *at the time of the study, only *Cx. quinquefasciatus *data were analysed and presented.

Of the 1018 female *Cx. quinquefasciatus *collected during the experimental hut trial, 22.5% (229/1018) had blood fed. The proportion of blood-fed *Cx. quinquefasciatus *was significantly lower in four treatment combinations when compared to the control treatment (Table 1). The level of blood feeding inhibition was similar for all three treatments that incorporated permethrin (CP, MP and BP). Of the fungus-only treatments, only the *B. bassiana *(BC) caused a significant reduction in the numbers of *Cx. quinquefasciatus *mosquitoes blood feeding (p = 0.032; Table 1). Although the level of blood feeding inhibition was similar for the permethrin (CP) and *B. bassiana *(BC) treatments, the combined *B. bassiana *and permethrin (BP) treatment showed no additive or synergistic effects of these two individual treatments (Table 1). The logistic regression analysis found no significant effect of the day, indicating that the levels of blood feeding did not significantly vary during the trial.

*Culex quinquefasciatus *mortality seven days after being collected from the huts was fairly similar for all six treatments (Table 1). Even when taking into account the variation between the replicates caused by doing the trial over a relatively long period of time, there was no significant impact of the *M. anisopliae *(HR = 1.03, p = 0.85), *B. bassiana *(HR = 1.12, p = 0.45) or permethrin ITN (HR = 1.02, p = 0.87) treatments on the mortality of mosquitoes when compared to the respective control treatments. Furthermore, there were no significant interactions between the fungal and insecticide treatments.

There was also no significant difference between the numbers of mosquitoes found in the huts for the six treatment combinations (F = 0.94, d.f. = 5,200, p = 0.46) indicating no significant deterrence of any of the treatments. The six treatment combinations also all had similar levels of induced exophiliy (F = 1.19, d.f. = 5,200, p = 0.32), and immediate mortality (the numbers of mosquitoes that were collected dead from the huts) (F = 0.35, d.f. = 5,200, p = 0.88).

## Discussion

This study was the first to examine the effect of entomopathogenic fungi on wild mosquito blood feeding in the field. In particular, the current study investigated more or less instantaneous impacts on feeding within a single feeding night (i.e. within a few hours of fungal exposure). The results show that *B. bassiana *treatments significantly reduced blood feeding, with *B. bassiana *alone able to inhibit 37% of blood feeding relative to the control. Permethrin was able to inhibit 43% of blood feeding, a higher percentage than observed in a previous study in the same study village where another pyrethroid, alphacypermethrin, reduced *Cx. quinquefasciatus *blood feeding by 27% [24]. Given the results, it is unknown why no additive or synergistic effects were seen in the blood feeding inhibitions when the *B. bassiana *(BC) and permethrin (CP) treatments were combined into the *B. bassiana *and permethrin (BP) treatment, especially in light of recent laboratory findings [21], although the synergistic effects seen on mortality [21] may differ from any behavioural effects.

Both of the fungal species used in our study have previously shown a propensity to reduce mosquito blood feeding under laboratory conditions [18,19]. This response may be linked to the down-regulation of genes controlling digestion in mosquitoes inoculated with *B. bassiana *[34] indicating that digestion and nutrient acquisition is not a priority for mosquitoes after fungal infection. Although these earlier studies looked at feeding over several days following infection and at different mosquito genera, they found a similar level of blood feeding reduction as found in our study [18,19]. Relatively rapid changes in feeding behaviour after infection with *M. anisopliae *or *B. bassiana *have also been reported in many other insect types [35].

The mechanism behind the very rapid blood feeding inhibition observed in the current study is unknown but may be due to physiological and/or behavioural reasons. A mosquito may enter the huts several hours before blood feeding which would allow the fungus time to start germination and cuticle penetration. As far as we are aware no data have been published on the germination and penetration times on mosquito cuticles. However, in infected termites *B. bassiana *germination occurred between 6 and 12 hours post infection, with penetration between 12 and 24 hours [36]. In this study, mosquitoes had to pass directly through the fungus-treated netting so some conidia could have got into the mosquito spiracles or at the base of the setae. This may decrease the fungal penetration time because the cuticle is thinner in these places [36]. Even during pre-penetration growth of the conidia the wax layer of the insect cuticle is degraded [37] and insects use both cellular and humoral immune responses against fungal infections starting as early as cuticle degradation [38]. Therefore, if the germination and pre-penetration times on mosquito cuticles is similar to that on termites, then it is feasible that the immune system could have been activated during the short time the mosquitoes and sleepers were in the huts. Alternatively, the mosquito antennae and maxillary palpi may have become covered in conidia, interfering with their ability to detect the human host. In addition, termites have been shown to groom after fungal infection which successfully removes conidia [39]. This may also have taken place with our wild mosquitoes and could have interfered with their host seeking.

The results indicate that neither *M. anisopliae *nor *B. bassiana *repels foraging mosquitoes, as corroborated by a recent laboratory study [40]. In addition, *B. bassiana *conidia can reduce blood feeding in *Cx. quinquefasciatus*. However, no significant mortality was found in wild-caught *Cx. quinquefasciatus *mosquitoes collected from the huts. Although previous findings have found that *Cx. quinquefasciatus *is susceptible to *M. anisopliae *[11,22] it is important to note that adult *Cx. quinquefasciatus *mortality has not previously been measured following *B. bassiana *infection either in the laboratory or field. Even after discounting the *M. anisopliae *data due to the extremely low viability of the batch used in our study, the *B. bassiana *viability was within the range that could be used in the field in the future, but did not significantly impact wild mosquito mortality. There are three main reasons why this may be the case.

Firstly, the experimental method may have been ineffective at providing a sufficiently lethal dose to the wild mosquitoes, even though it was able to elicit a significant behaviour modification. Possible reasons include certain conditions affecting the conidia on the netting, and the short contact time of the mosquitoes. After one week under field conditions dry conidia were seen to be released from the window netting in the huts. This quick evaporation of ShellSol T and release of conidia has also been found in Tanzania (Matt Kirby, Pers. Comm.) and may lead to a lack of conidial protection from the field conditions, and a decrease in the effective concentration. Using other oil formulations [22] or encapsulation techniques may lead to higher conidial protection. Laboratory studies have shown that conidial viability is directly affected by the polyester netting [15,41]. In addition, temperature and humidity can adversely affect fungal conidia [42-44], however, the climatic conditions were similar for the experimental hut study and cone bioassay experiments carried out at the same time and under the same conditions (Howard et al. Manuscript Submitted), so similar adverse effects would be expected. Nevertheless, fungal spores used in the cone bioassays were able to infect mosquitoes causing significant mortality (Howard et al. Manuscript Submitted), but those applied in the experimental huts could not.

Scholte et al. [22] found much higher levels of mortality in their field study where the *An. gambiae s.l. *mosquitoes were found resting on fungus-impregnated cotton cloths [22]. The short contact time with the hut fungal netting, although not an issue for *An. gambiae s.s. *mosquitoes in the behaviour experiments in the laboratory, could have caused problems because a recent study has shown that longer exposure times can cause significantly quicker mortality rates [45]. There appears to be a threshold number of conidia per unit surface area required for successful mosquito infection [11]. This may be related to the up-regulated mosquito immune system being able to clear low-level fungal infections [34,46]. If the proportion of viable conidia was decreased by the polyester netting/field conditions then the wild *Culex *may not have been receiving enough viable conidia to initiate a successfully lethal fungal infection. Other proposed application methods in the field include cotton resting targets [22], clay pots [17], and odour baited stations [23], all of which will ensure longer contact times but would target resting mosquitoes post-feeding, and so may not affect blood feeding in the same way as the method used in this study.

The second reason for the lack of fungus-induced mortality could be that even if a successful fungal infection was received by the hut-entering mosquitoes, then it is possible that the mosquitoes died of natural causes before any significant toxic effects of fungal infections could be seen because control mortality after 7 days was 54% and was not significantly different from the fungus and/or permethrin-exposed mosquitoes. This may have masked any effects of the fungus. The natural mortality could be quite high because the mosquitoes entering the huts were of an unknown age range, and insecticide resistance in *Culex *mosquitoes is known to be associated with fitness costs [47] that can lead to reduced survival rates [48].

Finally, the third possible reason for the lack of fungal-induced mortality is that the wild multi-insecticide-resistant *Cx. quinquefasciatus *mosquitoes in Benin may just not have been susceptible to fungal infection. As mentioned, *Cx. quinquefasciatus *adults have not been previously shown to be susceptible to *B. bassiana *either in the laboratory or field. A previous laboratory study comparing *An. gambiae s.s. *and *Cx. quinquefasciatus *found very few differences in susceptibility to *M. anisopliae *infection, with both male and female *Cx. quinquefasciatus *having significantly reduced life spans after continuous exposure to both dry and oil-formulated conidia [11]. However, Scholte [49] speculates that wild Tanzanian insecticide-susceptible *Cx. quinquefasciatus *in the field had higher immunocompetence towards *M. anisopliae *infection than wild *An. gambiae s.l. *because the infection rates were 10% and 33% respectively. Wild *Culex *may be less susceptible to fungal infection due to interactions of their micro-flora [50], or because their insecticide resistance mechanisms protected them [51]. Micro-flora interactions can protect insects from infection; *Pseudomonas *bacteria found in insecticide-resistant diamond-back moths showed antagonistic activity against *M. anisopliae *and *B. bassiana *[50]. In addition, *B. bassiana *has been shown to be a poor competitor in the presence of *M. anisopliae *[45], and is therefore likely to compete poorly with the wild *Culex *gut flora. Insecticide-resistant *Cx. quinquefasciatus *in Sri Lanka were shown to adversely affect the development of the filarial worm *Wuchereria bancrofti*, thought to be due to elevated esterase activity [51]. Serebrov et al. [52] found that infection of greater wax moth caterpillars with *M. anisopliae *caused elevated levels of esterases and GST, presumably as part of the immune response. If elevated esterase and GST levels are also an important immune response to fungal infection in *Cx. quinquefasciatus*, this would explain the low susceptibility of the wild mosquitoes in this study; in effect their immune system is already activated because they naturally have higher levels of these enzymes [3]. Further work should be carried out to identify whether wild insecticide-resistant *Culex *mosquitoes can be killed using entomopathogenic fungi, and to identify for which reason this did not occur in the present study.

Although there were not enough malaria vectors to analyse the effect the fungi may have on these mosquitoes, it is also important to test new control tools on *Culex *mosquitoes. This is because in many areas *Culex *mosquitoes are often more numerous than *Anopheles *and as such personal protection methods such as ITNs are often bought to prevent the nuisance biting as much as for any other reason. Failure to control these nuisance mosquitoes can reduce the uptake of ITNs for malaria control [53,54]. Therefore tools that can reduce the biting of insecticide-resistant *Culex *mosquitoes are also required. This is especially true in East Africa, India and South East Asia where *Culex *mosquitoes are the vectors of filariasis.

Reducing blood feeding is important in terms of disease control and the finding that *B. bassiana *can reduce blood feeding in wild mosquitoes so soon after they acquired a fungal infection is both unexpected and important, but further research in areas with filariasis transmission is required to monitor whether this behaviour modification could be used to help prevent filariasis transmission. In addition, work should also be carried out specifically targeting malaria vectors to substantiate whether this behaviour is also present in *Anopheles *mosquitoes, and if this could have any effect on malaria transmission. Because blood feeding was significantly affected so soon after acquiring a fungal infection it is suggested that future application techniques for fungi in the field should target host-seeking mosquitoes. If the fungi are deployed as post-feeding resting targets [17,22,23], then one of the main ways in which entomopathogenic fungi could help reduce disease transmission would be missed.

## Competing interests

The authors declare that they have no competing interests.

## Authors' contributions

AFVH helped to design the study, undertook the data collection, analysed part of the data and drafted the manuscript. RN'G aided in study design and coordinated data collection. CJMK analysed part of the data. AA aided in data collection. MF aided in study design. MA coordinated supervision in Benin. MBT aided in drafting the manuscript. BGJK conceived of the study, and participated in its design and supervision. WT supervised the study and aided in drafting the manuscript. All authors read and approved the final manuscript.
